# Disparities in Surgical Intervention in Pediatric Patients With Submucous Cleft Palate and Velopharyngeal Dysfunction

**DOI:** 10.1002/ohn.1111

**Published:** 2025-01-07

**Authors:** Soukaina Eljamri, Pooja D. Reddy, Amber Shaffer, Randall J. Harley, Noel Jabbour

**Affiliations:** ^1^ Department of Otolaryngology University of Pittsburgh School of Medicine Pittsburgh Pennsylvania USA; ^2^ Department of Pediatric Otolaryngology UPMC Children's Hospital of Pittsburgh Pittsburgh Pennsylvania USA; ^3^ Department of Otolaryngology University of Pennsylvania Philadelphia Pennsylvania USA

**Keywords:** area deprivation index, child opportunity index, disparities, submucous cleft palate, velopharyngeal dysfunction

## Abstract

**Objective:**

To evaluate factors impacting access to and timing of surgery in patients with submucous cleft palate (SMCP) and velopharyngeal dysfunction (VPD).

**Study Design:**

Retrospective cohort study.

**Setting:**

Single academic medical center.

**Methods:**

Patients with SMCP and VPD between 2004 and 2021 were identified. Variables included national and state area deprivation index (ADI) percentiles, child opportunity index (COI) categories, distance to care, and insurance status. *χ* ^2^, Fisher's exact test, Wilcoxon rank‐sum, Spearman rank correlation, *t* test, and linear regression (*α* = .05) were used to investigate the relationships between these variables and surgical status and timing.

**Results:**

A total of 168 patients were included, 94 surgical and 74 nonsurgical. Patients were predominantly white (160/168; 95.2%), Male (103/168; 61.3%), and non‐Hispanic (153/168; 91.1%). There were no intergroup differences with respect to ADI, COI, insurance status, or distance from the hospital. Surgical patients were more likely to have overt SMCP (*P* = .03), earlier age at SMCP diagnosis (*P* = .02), and higher baseline Pittsburgh weighted speech score (PWSS) (*P* = <.001). In multivariable regression, younger age at surgery was found to be significantly associated with higher baseline PWSS (*P* = .001) and lower state ADI deciles (*P* = .03). Patients with private insurance had a lower baseline PWSS than those with public insurance (*P* = .04). Insurance status was not significantly associated with age at diagnosis (*P* = .79) or age at surgery (*P* = .08).

**Conclusion:**

In this study, patients from less disadvantaged neighborhoods were found to have earlier surgical intervention, highlighting the importance of incorporating social determinants of health in the evaluation of VPD and SMCP patients to prevent treatment delays.

Submucous cleft palate (SMCP) involves impaired median fusion of the muscles of the soft palate and is characterized by Calnan's triad: zona pellucida, bifid uvula, and notching of the hard palate.^1^ There is a spectrum of severity in SMCP, requiring subclassification as overt or occult. An overt SMCP has 1 or more of the triad features, while occult has similar palatal muscle diastasis in the absence of the classic triad features. Approximately half of all SMCP patients are asymptomatic, while the other half may present with speech deficits and/or middle ear disease.^2^ Velopharyngeal dysfunction (VPD) is a consequence of SMCP that is defined by inadequate closure of the soft palate and pharynx to separate the oral and nasal cavities, resulting in hypernasal speech. Surgical repair of SMCP is usually indicated for correction of these speech deficits.^2^, ^3^ While the American Cleft Palate‐Craniofacial Association provides specific guidelines on the optimal timing for cleft repair, the recommendations for timing of SMCP repair are less clear given the tremendous variability in patient presentation.^4^

Multiple studies have demonstrated that patients with earlier correction of cleft palate are less likely to develop VPD at a later age.^5^, ^6^ Further, delayed correction of both submucous and overt cleft palates has been associated with an increased incidence of articulation errors and persistent misarticulation.^7^ Numerous factors, including socioeconomic variables and structural barriers, may impact whether patients undergo surgical correction of SMCP and the subsequent timing of their diagnosis and intervention. A recent study of 260 patients undergoing surgical repair for cleft palate found that black, Asian Pacific Islander, Spanish‐speaking patients, as well as those traveling a greater distance to the hospital, had delayed cleft palate repairs, ranging from 11 to 52 days of delay.^5^ A similar study of 135 patients with cleft lip and/or palate found that surgical repair was delayed in African‐American and Asian‐American Pacific Islander patients.^6^ A large database study of over 6000 patients who underwent primary cleft lip and/or cleft palate repair found that non‐white children had lower rates of repair overall compared to white children. Further, Asian children in this study had the highest rates of delayed surgical repair, while black and Hispanic children had higher rates of readmission following cleft repair.^7^

These findings reflect much of what has been established across specialties, where disparities in health access and outcomes disproportionally impact non‐white and non‐English speaking populations. Given the importance of timely intervention to prevent speech deficits, in this study, we aimed to identify disparities in the timing of surgical repair in patients with SMCP and VPD, using both individual and composite measures of deprivation.

## Materials and Methods

This study was reviewed and approved by the University of Pittsburgh Institutional Review Board (STUDY19100247). A retrospective chart review was conducted of patients with SMCP and VPD from a single tertiary pediatric hospital, who were diagnosed with SMCP between 2004 and 2021. Primary outcomes included age at SMCP diagnosis and time between diagnosis and surgery among surgical patients. Covariates included sex, race, SMCP severity (overt vs occult), baseline Pittsburgh weighed speech score (PWSS), area deprivation index (ADI) national percentiles and state deciles, child opportunity index (COI) categories, distance to the pediatric hospital (miles), and insurance status (private vs public). As pediatric patients with certain disabilities may qualify for public medical assistance regardless of family income, patients were categorized as having public insurance when they did not have any secondary private coverage. Insurance status was determined based on the date of SMCP diagnosis for both surgical and nonsurgical patients.

The Pittsburgh weighted speech score is a validated measure to evaluate VPD on a quantitative scale, using the following categories: nasal air emission, presence of facial grimace, nasality/resonance, phonation/voice, and articulation. The scoring guidelines are as follows: scores of 1 to 2 indicate borderline competence, scores of 3 to 6 indicate borderline incompetence, and scores greater than 7 indicate velopharyngeal incompetence.^8^

ADI state deciles and national percentiles were obtained using each patient's full address with a 9‐digit zip code using the online Neighborhood Atlas database. ADI data is publicly available for 2022, 2020, and 2015, with each year's database capturing a 5‐year period: 2018 to 2022, 2016 to 2020, and 2011 to 2015, respectively.^9^ ADI metrics were assigned to each patient according to the available ADI year closest to their year of SMCP diagnosis. National percentiles ranged from 1 (lowest disadvantage) to 100 (highest disadvantage), and state deciles ranged from 1 to 10 using a similar scale. COI scores were retrieved from the diversitydatakids.org database using the following data controls: zip codes and national level of comparison. COI data is available for the years 2012, 2017, and 2021. As with ADI data, each patient was assigned a COI year that was closest to their year of SMCP diagnosis.^10^ Scores were obtained using the patient's full address with a 9‐digit zip code and patients were categorized into 1 of the following groups: very low, low, moderate, high, and very high.

*χ* ^2^, Fisher's exact test, Wilcoxon rank‐sum, Spearman rank correlation, *t* test, and linear regression (*α* = .05) were used to investigate the relationships between these variables, surgical status, and time to surgery. To determine factors impacting age at surgery, a multivariable linear regression model was constructed, using PWSS and ADI national percentile as predictors and age at surgery as the response. Stata/SE 16.1 (StataCorp) was used for analysis.

## Results

### Demographics

168 patients were included in this study, 94 surgical and 74 nonsurgical. They were predominantly white (160/168; 95.2%), male (103/168; 61.3%), and non‐Hispanic (153/168; 91.1%). In terms of SMCP severity, most patients had occult SMCP (103/168; 61.3%) and a median baseline PWSS of 8 (interquartile range [IQR]: 4‐16). The median age at SMCP diagnosis was 4.5 years (IQR: 3.1‐6.5). Patients had a median household income of $68,069 (IQR: $55,903‐$91,445) and resided a median distance of 27.9 miles from UPMC Children's Hospital of Pittsburgh (IQR: 13.8‐57.3 miles). Most patients had private insurance (102/168; 60.7%), and there was no significant difference in insurance status between surgical and nonsurgical patients (*P* = .5) (Table 1).

**Table 1:** Patient Characteristics

	Overall	Nonsurgical	Surgical	
	n (%)	n (%)	n (%)	*P*
Female	65/168 (38.7%)	23/74 (31.1%)	42/94 (44.7%)	.07
Race				
White	160/168 (95.2%)	72/74 (97.3%)	88/94 (93.6%)	
Black	6/168 (3.6%)	2/74 (2.7%)	4/94 (4.3%)	.7
Asian	2/168 (1.2%)	0/74 (0.0%)	2/94 (2.1%)	
Ethnicity				
Non‐Hispanic	153/168 (91.1%)	69/74 (93.2%)	84/94 (89.4%)	.1
Hispanic	3/168 (1.8%)	3/74 (4.1%)	0/94 (0.0%)	
Not reported	12/168 (7.1%)	2/74 (2.7%)	10/94 (10.6%)	
Child opportunity index				.9
Very low	15/168 (8.9%)	4/74 (5.4%)	11/94 (11.7%)	
Low	36/168 (21.4%)	19/74 (25.7%)	17/94 (18.1%)	
Moderate	47/168 (28.0%)	21/74 (28.4%)	26/94 (27.7%)	
High	41/168 (24.4%)	19/74 (25.7%)	22/94 (23.4%)	
Very high	29/168 (17.3%)	11/74 (14.9%)	18/94 (19.2%)	
Private insurance	102/168 (60.7%)	47/74 (63.5%)	55/94 (58.5%)	.5
Overt SMCP	65/168 (38.7%)	22/74 (29.7%)	43/94 (45.7%)	**.03**

	Median (IQR)	Median (IQR)	Median (IQR)	*P*
Area deprivation index (state)	6 (3‐8)	6.5 (3‐9)	6 (3‐8)	.1
Area deprivation index (national)	62 (41‐82)	69 (42‐85)	60 (41‐76)	.06
Median household income, $	68,069 (55,903‐91,445)	62,491 (54,548‐92,480)	70,217 (57,377‐91,445)	.2
Age at SMCP diagnosis, y	4.5 (3.1‐6.5)	5.8 (2.9‐7.6)	4.1 (3.1‐5.1)	**.02**
Baseline PWSS	8 (4‐16)	4 (1‐5)	14 (9‐21)	**<.001**
Distance to hospital, miles	27.9 (13.8‐57.3)	32.1 (15.7‐54.9)	26.4 (11.7‐59.2)	.6

Bold indicates *P* < .05.

Abbreviations: IQR, interquartile range; PWSS, Pittsburgh weighted speech score; SMCP, submucous cleft palate.

### Composite Deprivation Measures

**Figure 1:**
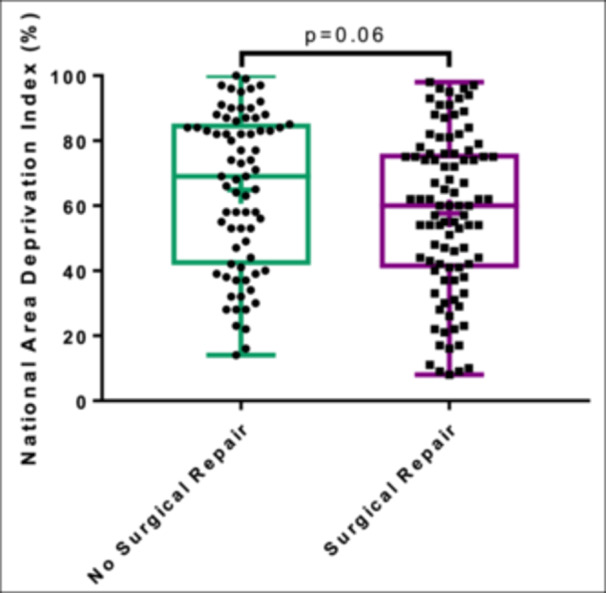
Surgical status and national ADI percentile. Box and whisker plot representation of national ADI percentile and surgical status, demonstrating no significant difference between surgically and nonsurgically managed patients (Wilcoxon rank‐sum *P* = .06) + indicates mean. ADI, area deprivation index.

**Figure 2:**
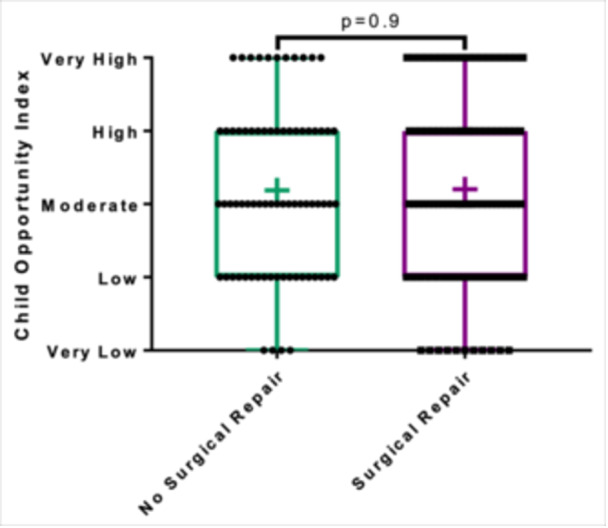
Surgical status and childhood opportunity index. box and whisker plot representation of childhood opportunity index classification and surgical status demonstrating no significant difference between surgically and nonsurgically managed patients (Wilcoxon rank‐sum *P* = .9) + indicates mean.

Patients had a median ADI state decile of 6 (IQR: 3‐8) and a national percentile of 62 (IQR: 41‐82). With regards to COI, 15 patients were in the very low category (8.9%), 36 were in the low category (21.4%), 47 in the moderate (28.0%), 41 in the high (24.4%), and 29 in the very high category (17.3%). There were no significant differences in ADI or COI between surgical and nonsurgical patients (*P* = .06 and *P* = .9, respectively) (Figures 1 and 2).

### Surgical Characteristics and Timing

**Figure 3:**
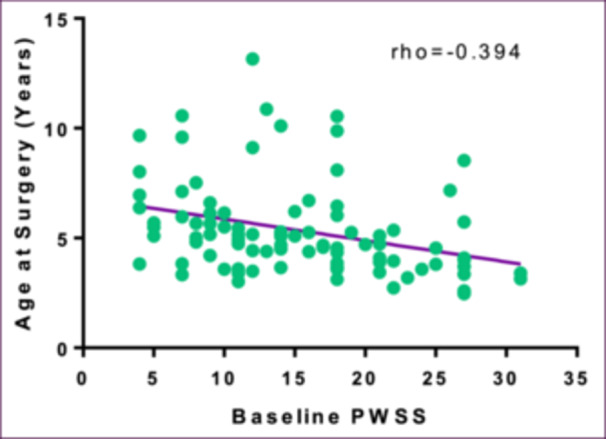
Age at surgery and baseline PWSS. Graphical representation of the univariate association between age at surgery and PWSS, demonstrating decreased age at surgery in years with increasing baseline PWSS (Spearman rank correlation *P* < .001). PWSS, Pittsburgh weighted speech score.

**Figure 4:**
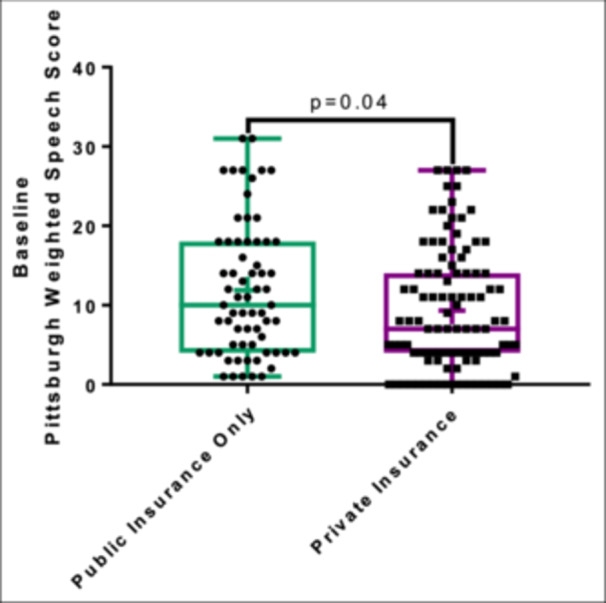
Insurance status and baseline PWSS. Box and whisker plot representation of insurance status and PWSS demonstrating that patients with private insurance had significantly lower baseline PWSS compared to those with public insurance only (Wilcoxon rank‐sum *P* = .04) + indicates mean. PWSS, Pittsburgh weighted speech score.

Most patients underwent surgical repair (94/168; 56.0%) and the predominant indication for surgery was speech concerns (92/94; 97.9%) (Table 2). Surgical patients were significantly more likely to have overt SMCP (*P* = .03), earlier age at SMCP diagnosis (*P* = .02), and higher baseline PWSS (*P* < .001) (Table 1). The median age at surgery was 5.0 years (IQR: 3.8‐6.0) and the time from diagnosis to surgery was 5.5 months (IQR: 3 months to 1.1 years) (Table 2). In multivariable linear regression, younger age at surgery was significantly associated with higher baseline PWSS (*P* = .001) and lower state ADI deciles (*P* = .03) (Figure 3, Table 3). Patients with private insurance had a lower baseline PWSS compared to those with public insurance (*P* = .04; median 7, IQR: 4‐14 vs median 10, IQR: 4‐18) (Figure 4). Insurance status was, however, not significantly associated with age at diagnosis (*P* = .8) or age at surgery (*P* = .08).

**Table 2:** Surgical Indications and Timing

	n (%)
Surgical repair	94/168 (56.0%)
Indication for surgery	
Fistula	0/94 (0.0%)
Speech	92/94 (97.9%)
Other	2/94 (2.1%)
	Median (IQR)
Time from diagnosis to surgery	5.5 months (IQR: 3 mo‐1.1 y)
Age at surgery, y	5.0 (IQR: 3.8‐6.0)

Abbreviation: IQR, interquartile range.

**Table 3:** Factors Impacting Age at Surgery

	*Β*	95% CI	*P*
Baseline Pittsburgh weighted speech score	−0.1055	−0.1625, −0.0484	<.001*
Area deprivation index (state)	0.1591	0.0119, 0.3063	.03*

Abbreviation: CI, confidence interval.

* *P* < .05.

## Discussion

Delayed surgical repair in patients with SMCP has been associated with permanent articulation errors and persistent speech deficits.^11^, ^12^, ^13^ Our study aimed to identify variables at both the individual and regional level that contribute to disparities in access to and timing of surgical repair in patients with SMCP and VPD. We demonstrated that patients who underwent surgical correction for SMCP were more likely to have overt SMCP, higher baseline PWSS, and earlier age at SMCP diagnosis. Interestingly, there were no significant differences between surgical and nonsurgical patients across ADI national percentiles and state deciles, COI categories, and insurance status, suggesting that these variables were less likely to contribute to the patient's surgical status. In terms of timing, younger age at surgery was found to be significantly associated with a higher baseline PWSS, indicative of more severe VPD, and lower state ADI deciles, suggesting that patients residing in less disadvantaged neighborhoods had earlier surgical intervention. Insurance status did not differ significantly between surgical and nonsurgical patients and was not associated with age at diagnosis or age at surgery. However, in our cohort, patients with private insurance had a lower baseline PWSS than those with public insurance.

Composite measures of deprivation are helpful indicators of systemic disparities on a regional scale. The ADI is a composite metric of 17 US Census variables including education, employment, income, poverty level, and household characteristics. The COI is a similar composite measure that incorporates 44 neighborhood‐level indicators, which are grouped into 3 overall domains: education, health and environment, and social and economic. These indicators are meant to reflect the experiences of a typical child in a given area of interest. While there is no current study evaluating the impact of neighborhood deprivation on surgical timing for SMCP repair, in a study examining the impact of neighborhood deprivation on outcomes, interventions, and length of follow‐up in 205 patients with cleft palate, the authors found that patients from more deprived ADI neighborhoods had worse speech and language development outcomes.^11^ Similarly, a recent study examining the sociodemographic factors contributing to delays in craniosynostosis surgery found that patients with public insurance and those who resided in a higher ADI decile, indicating greater disadvantage, had an older age of presentation and delayed surgical correction.^12^ In an effort to promote equitable and timely health care access, it is important to understand how regional deprivation and resource limitations may impact patients' ability to receive care.

Insurance status has been previously associated with delays in both diagnosis and treatment of SMCP, specifically in patients with public insurance.^14^ In our study, insurance status was not correlated with surgical status or timing of diagnosis or surgery. However, patients with private insurance had a lower baseline PWSS than those with public insurance, suggesting that individuals with private insurance may have had a lower threshold for recognition of speech deficits and were thus less symptomatic at the time of referral. Timely diagnosis and treatment of cleft care are also dependent on timely recognition and referral to specialists, which is largely driven by primary care providers. The differences in primary care afforded to those with private insurance may drive differences in these referral patterns. A recent study found an improvement in socioeconomic disparities in cleft care, including a decrease in age at presentation, with the implementation of a “cleft nurse navigator program.” In this model, a registered nurse served as a liaison between families and the cleft team from the prenatal period through adolescence and facilitated early symptom recognition and referral to care.^15^ Considering similar interventions for patients with SMCP may assist in preventing unnecessary delays.

Similarly, distance to care has also been correlated with delays in the diagnosis and management of cleft patients.^5^ In our study, distance from the hospital was not significantly associated with age at diagnosis or surgical timing. However, our cohort had a median distance of 27.9 miles (IQR: 13.8‐57.3) from the care center, which is relatively proximal compared to more rural regions, where patients may have a longer median distance to care. For context, the UPMC Children's Hospital of Pittsburgh Cleft and Craniofacial Center is 1 of 3 centers within a 100‐mile radius, with the WVU Medicine Cleft & Craniofacial Center and the James A. Lehman Jr, MD, Craniofacial Center at Akron Children's being the next closest regional providers. In a study investigating delays in the treatment of cleft palate in 260 patients, significantly greater age at the time of surgery was seen in patients who had traveled over 360 miles to care. Notably, patients in this study had a median distance from the hospital of 23.2 miles with a range of 0 to 7780 miles.^5^ While distance to the hospital was not correlated with surgical timing in our cohort, we found that patients from lower ADI neighborhoods had earlier surgical intervention. Of the 17 variables incorporated in the ADI, only 1 is “percentage of occupied housing units without a motor vehicle.”^9^ These findings together highlight the multifaceted nature of socioeconomic disparities and underscore the importance of analyzing these disparities with composite measures to better capture this complexity. A study on sociodemographic factors associated with delayed presentation in craniosynostosis patients similarly found that ADI deciles did not correlate with distance to care. With this finding, the authors emphasized that financial resources may be more predictive of access to care than distance.^16^ In other words, even further distances to care may be more easily navigated with adequate financial resources.

Our study has important limitations. This is a retrospective study with a limited sample size. Our patients were predominantly white, male, and non‐Hispanic, thus generalizability is limited. Future studies with a more diverse study population may improve the generalizability of these findings. Further, given the limited ADI and COI data available, these metrics could not be directly correlated with the exact year of SMCP diagnosis for all patients, thus only data available in the year closest to the year of diagnosis was used. Future prospective studies may capture these composite measures with more accurate timing.

## Conclusion

The timing of diagnosis and surgical intervention in patients with SMCP and VPD is associated with long‐term outcomes in speech development, with earlier intervention yielding better long‐term outcomes during the critical window for speech development. Among all studied variables, only PWSS and ADI were significantly associated with surgical timing, with younger age at surgery seen in patients with higher baseline PWSS and lower state ADI deciles. These findings indicate that symptom recognition and surgical timing in patients with VPD may be impacted by socioeconomic and structural variables. Incorporating social determinants of health into the initial evaluation of patients with SMCP and VPD may assist in predicting barriers and enhancing timely access to treatment.

## Author Contributions

**Soukaina Eljamri**, conceptualization, data curation, investigation, methodology, writing—original draft preparation, review, and editing; **Pooja D. Reddy**, conceptualization, data curation, writing—review and editing; **Amber Shaffer**, conceptualization, formal analysis, methodology, writing—review and editing; **Randall J. Harley,** conceptualization, methodology, writing—review and editing. **Noel Jabbour**, conceptualization, methodology, project administration, supervision, writing—review and editing.

## Disclosures

### Competing interests

The authors have no conflicts of interest to declare.

### Funding source

The authors have no financial disclosures.
